# Veterinary Register

**Published:** 1901-12

**Authors:** 


### VETERINARY REGISTER.

[See Editorial, Journal of Comparative Medicine and Veterinary Archives, July, 1901, page 438.]

[In the following list the names and data printed without comment are those furnished to us by postal card with the signature of the individual.

Those with an asterisk [*] are those to whom we have sent, by United States mail, an envelope with prepaid postage and a request for return if not delivered in ten days ; as the envelopes have not been returned, we presume that the letter has been delivered, that the individual in question exists, but that he has neglected to return the enclosed postal card with the information requested.—ED. JOURNAL.]

### PENNSYLVANIA.

(Continued from page 752.)

| *Name.* | *Street.* | *Town.* | *College, Graduated.* | *County, Registered.* |
|---|---|---|---|---|
| Madden, Festus | ...... | Pittsburg | * | ...... |
| Magee, C. W. | ...... | Uniontown | * | ...... |
| Magee, George | ...... | Uniontown | * | ...... |
| Magill, Chalkley H. | 2032 N. 16th st | Philadelphia | V. D. U. Pa., 1889 | Philadelphia |
| Maher, John J. | 1336 Marshall | Philadelphia | V. D. U. Pa., 1890 | Philadelphia, 1890 |
| Manges, Abram B. | ...... | Helixville | * | ...... |
| Manley, W. S. | 207 N. 4th st | Philadelphia | * | ...... |
| Mansbach, L. A. | 915 N. 6th st | Philadelphia | * | ...... |
| Markley, G. W. | ...... | Mechanicsburg | * | ...... |
| Marlin, Edgar R. | 4537 Frkf'd av | Philadelphia | * | ...... |
| Marshall, C. J. | 2004 Pine st | Philadelphia | V. D. U. Pa., 1894 | Philadelphia |
| Marshall, James A. | 916 Brown st | Philadelphia | Pa. Vet. Surg., 1868 | Philadelphia, 1889 |
| Martien, Henry D. | 4247 Fairm't | Philadelphia | V. D. U. Pa., 1896 | Philadelphia, 1897 |
| Martin, | ...... | Mercersburg | * | ...... |
| Martin, David | 1218 Walnut st | McKeesport | * | ...... |
| Martin, David G. | ...... | New Holland | * | ...... |
| Martin, Harry | ...... | Elgin | * | ...... |
| Martin, Samuel H. | ...... | E. Earl | * | ...... |
| Martin, W. M. | 1831 Chestnut | Philadelphia | * | ...... |
| Mase, John | ...... | Sebring | * | ...... |
| Masiker, Moses | ...... | Randolph | * | ...... |
| Massinger, Chas. | ...... | Chalfont | * | ...... |
| Massinger, E. M. | 211 Morgan st | Phoenixville | N. Y. C. V. S., 1891 | Bucks, 1891<br>Chester, 1893 |
| Massinger, Wesley | ...... | Chalfont | * | ...... |
| Mather, John W. | ...... | Rohrsburg | * | ...... |
| Mathues, Samuel W. | ...... | Concordville | N. Y. C V. S., 1889 | Delaware, 1889 |
| Mattson, Wm. H. | ...... | Chester H'ts | V. D. U. Pa., 1889 | Delaware, 1889 |
| Mauch, John H. | ...... | Vicksburg | * | ...... |
| Mauser, N. | ...... | Mifflinburg | * | ...... |
| Mauser, Noah | ...... | Mifflinburg | * | ...... |
| May, A. R. | ...... | Boiling Sp'gs | ...... | Cumberland, 1889 |
| May, Reuben | ...... | York Springs | * | ...... |
| Mayer, Henry W. | 4722 Liberty av | Pittsburg | * | ...... |
| Mays, John B. | ...... | Jordan | * | ...... |
| Meade, L. E. | Bridge st | Tunkhannock | National Vet., 1894 | Wyoming, 1894 |
| Mearig, Israel K. | ...... | Leacock | * | ...... |
| Meckley, Urius L. | ...... | Hanover | * | ...... |
| Megargee, Franklin | ...... | Coatesville | * | ...... |
| Mellor, Austin | ...... | Philadelphia | * | ...... |
| Mengee, Fred | ...... | Germania | * | ...... |
| Meredith, W. A. | 62 N. Centre st | Corry | Ontario Vet., 1885 | Erie, 1889 |
| Merian, John | ...... | Pittsburg | * | ...... |
| Mertz, P. J. | ...... | Honeybrook | Ontario Vet., 1895 | Chester, 1895 |
| Mertz, Rudolph | 448 Schuylkill | Reading | Ohio Vet. Med., 1894 | Berks, 1894 |
| Messinger, David | ...... | Hampden | * | ...... |

| *Name.* | *Street.* | *Town.* | *College, Graduated.* | *County, Registered.* |
|---|---|---|---|---|
| Meyer, G. | Sandusky st & Church av | Allegheny | * | ...... |
| Meyer, S. W. | ...... | Shippensburg | * | ...... |
| Michener, E. M. | ...... | Hatfield | * | ...... |
| Michener, E. M. | 128 W. Main st | North Wales | V.D. U. Pa., 1890 | Montgomery, 1890 |
| Michener, J. Curtis | ...... | Colmar | * | ...... |
| Milleisen, A. W. | ...... | Mechanicsb'g | * | ...... |
| Miller, Chas. | ...... | Duncannon | * | ...... |
| Miller, D. H. | ...... | Allentown | * | ...... |
| Miller, David W. | ...... | Lewisburg | * | ...... |
| Miller, Franklin W. | ...... | Gouglersville | * | ...... |
| Miller, H. H. | ...... | Rohrsburg | * | ...... |
| Miller, Henry | ...... | Canonsburg | * | ...... |
| Miller, Jacob | ...... | Mountainville | * | ...... |
| Miller, Jared | ...... | Tulpehocken | * | ...... |
| Miller, Joel | 2929 Frkf'd av | Philadelphia | * | ...... |
| Miller, John P. | 46 Reed st | Reading | V.D. U. Pa., 1899 | Berks, Lebanon, 1899 |
| Miller, Joseph | ...... | Rebersburg | * | ...... |
| Miller, Samuel H. | ...... | Blueball | * | ...... |
| Miller, W. T. | Armstrong av | Apollo | American Vet., 1887 | Indiana, 1889 |
| Mincum, Elias | ...... | Middleburg | * | ...... |
| Minick, B. F. | 424 Walnut st | Columbia | * | ...... |
| Minster, P. M. | ...... | Langhorne | * | ...... |
| Mitchell, A. J. | 1237 Peach st | Erie | American Vet. Ontario Vet. | Erie |
| Mock, Franklin J. | ...... | Loveville | * | ...... |
| Mock, W. E. | 544 Wilkesb're | Easton | * | ...... |
| Mogel, Emendon | ...... | Bernville | * | ...... |
| Mohr, John H. | ...... | Allentown | * | ...... |
| Montgomery, Alex. | ...... | Kittanning | * | ...... |
| Montgomery, A. H. | ...... | Bryn Mawr | * | ...... |
| Montgomery, H. E. | ...... | Kittanning | * | ...... |
| Montgomery, W. B. | Rex av | Chestn't Hill | V. D. U. Pa., 1887 | Philadelphia |
| Moor, Edward | ...... | Lima | * | ...... |
| Moore, Enoch H. | 1328 S. 8th st | Philadelphia | * | ...... |
| Moore, J. W. | ...... | Waynesboro | * | ...... |
| Morgan, W. D. | ...... | Plymouth | * | ...... |
| Moriarty, M. | Chambersb'g st | Gettysburg | ...... | York, 1889 |
| Morris, Franklin | ...... | Bucksville | American Vet., 1901 | Bucks, 1901 |
| Morrison, W. H. | Main st. | Holmesburg | * | ...... |
| Morrow, Jacob W. | 527 Lancaster | Lancaster | ...... | Lancaster, 1889 |
| Moss, Mahlon H. | ...... | Bristol | * | ...... |
| Moyer, Enos S. | ...... | Bloom'g Glen | N. Y. C. V. S., 1892 | Bucks, 1892 |
| Moyer, Henry B. | ...... | Huff's Church | * | ...... |
| Moyer, Simon S. | ...... | Hilltown | * | ...... |
| Moyer, Theo. W. | ...... | Siegfried | American Vet., 1886 | North'mpton, 1894 |
| Moyer, Wm. H. | ...... | Mechanicsb'g | * | ...... |
| Muir, Edwin Stanton | 2145 N. 2d st | Philadelphia | V. D. U. Pa., 1890 | Philadelphia, 1890 |
| Mumma, R. T. | ...... | Hanover | National Vet., 1895 | Adams, 1895 |
| Munger, F. W. | ...... | Beaver | * | ...... |
| Munchland, H. J. | ...... | Eldersville | * | ...... |
| Murphy, Patrick F. | 1811 N. 2d st | Philadelphia | * | ...... |
| Murphy, Wm. | ...... | W. Fairfield | * | ...... |
| Myers, Christian | ...... | Carversville | * | ...... |
| Myers, Esau C. | ...... | Mt. Pleasant | * | ...... |
| Myers, George | ...... | Dover | * | ...... |
| Myers, Henry C. | ...... | Buckingham | * | ...... |
| Myers, Thomas | ...... | Oxford | * | ...... |
| McAnulty, Jas. T. | 1445 S. 8th st | Philadelphia | ...... | Philadelphia, 1889 |
| McAnulty, John F. | 2832 N. 6th st | Philadelphia | V. D. U. Pa., 1898 | ...... |
| McCandless, Greer | ...... | Conoquen'ing | * | ...... |
| McCarthy, F. J. | ...... | Pottsville | * | ...... |
| McCarty, Edward | 100 Jackson st | Philadelphia | * | ...... |
| McClatchey Chas. | ...... | Slate Lick | * | ...... |
| McClintock, Harry T. | ...... | Milton | * | ...... |
| McColey, Thomas | ...... | Carnegie | * | ...... |

| *Name.* | *Street,* | *Town.* | *College, Graduated.* | *County, Registered.* |
|---|---|---|---|---|
| McCourt, James | ...... | Philadelphia | * | ...... |
| McCoy, W. E. | ...... | Oil City | * | ...... |
| McCoy, Wm. J | 3413 Kipp st | Philadelphia | Pa. C. V. S., 1885 | Philadelphia, 1889 |
| McCray, W. E. | 229 Seneca st | Oil City | * | ...... |
| McFadden, Jacob | ...... | Clintonville | * | ...... |
| McFayden, John | ...... | Chester | * | ...... |
| McGary, J. J. | ...... | Gallitzin | * | ...... |
| McGeary, J. J. | ...... | Gallitzin | * | ...... |
| McGranaham, W. S. | ...... | Sheakleyville | * | ...... |
| McGugan, James | 1209 S. 7th st | Philadelphia | * | ...... |
| McInally, J. G. | ...... | Lyn Valley | * | ...... |
| McKeever, Arthur | 511 S. 2d st | Philadelphia | * | ...... |
| McKenna, Charles S. | ...... | Kane | * | ...... |
| McKibbin, Robt. W. | ...... | Warfordsb'g | Ontario Vet., 1895 | Fulton, 1897 |
| McKinley, S. R. | ...... | Elk Lick | * | ...... |
| McKissick, N. G. | ...... | Millersburg | * | ...... |
| McLean, Charles C. | 1001 Water st | Meadville | Ontario Vet., 1883 | Crawford, 1889 |
| McLean, John | ...... | Meadville | * | ...... |
| McMillen, Wm. | ...... | S. Mahoning | * | ...... |
| McMullen, Hugh | ...... | Conneautville | * | ...... |
| McMullen, S. F. | ...... | Beaver Centre | * | ...... |
| McNeal, Frank J. | ...... | Shickshinny | * | ...... |
| McNeal, H. S. | ...... | Shickshinny | * | ...... |
| McNeal, Harry T. | 17 Elm st | Milton | Ontario Vet., 1895 | Northumb'd, 1889 Luzerne, 1895 |
| McNeal, Pete | Centre ave | Plymouth | Ontario Vet., 1896 | Luzerne, |
| McNees, John M. | ...... | W. Liberty | * | ...... |
| McNees, J. S. | ...... | W. Liberty | * | ...... |
| McNeil, Jas. C. | Dept. Public Safety | Pittsburg | * | ...... |
| Nagle, Geo. | ...... | Uniontown | * | ...... |
| Napp, Nelson | ...... | Pinegrove | * | ...... |
| Neff, Christian | ...... | Port Royal | * | ...... |
| Neff, Wm. H. C. | 2136 G'twn av | Philadelphia | * | ...... |
| Nester, Abraham | ...... | New Hanover | * | ...... |
| Nester, Jacob | ...... | New Hanover | * | ...... |
| Newcomer, Christian | ...... | Woodbury | * | ...... |
| Newcomer, Ezra W. | ...... | Mt. Joy | V. D. Univ. Pa., 1899 | Lancaster, 1899 |
| Newhard, Irwin C. | Centre st | Ashland | N. Y. C. V. S., 1893 | Dauphin, 1893 Schuylkill, 1896 |
| Nibel, Chas. B., | ...... | Bowmans'lle | * | ...... |
| Nice, Frank K. | Tioga V. Hosp. 1522-26 Erie av | Philadelphia | * | ...... |
| Nice, Wm. H. | Morton and Hancock st | Gtn., Phila. | * | ...... |
| Nicholas, W. S. | ...... | Bedmins'lle | * | ...... |
| Nichols, S. | ...... | Wellsboro | Ontario Vet., 1887 | Tioga, 1892 |
| Nicholson, Sam'l J. | 3061 Richmond | Philadelphia | V. D. Univ. Pa., 1892 | Philadelphia, 1892 |
| Nicholson, Wm. | 85 Taylor av | Allegheny | * | ...... |
| Nick, Chas. W. | ...... | McKean | * | ...... |
| Niles, W. S. | Main and Lookout sts | Pleasant Mt. | Ontario Vet., 1889 | Wayne, 1890 |
| Noack, Otto G. | 54 S. 6th st | Reading | Berlin, 1890 | Berks, 1893 |
| Oat, Chas F. | ...... | West Chester | * | ...... |
| O'Donnel, David A. | 36 Lehman st | York | ...... | York, 1890 |
| O'Donnell, M. J. | ...... | St Clair | * | ...... |
| Oechsle, Jos. | 2947 Girard av | Philadelphia | * | ...... |
| Ohe, Daniel | ...... | Penn | * | ...... |
| Ohly, A H. | 50 Avery st | Allegheny | * | ...... |
| O'Kane, Patrick | 1634 Barker st | Philadelphia | * | ...... |
| O'Laughlin, C. | ...... | Allegheny | * | ...... |
| Olin, Oneor | ...... | Jackson | * | ...... |
| Olweiler, Jacob F. | ...... | Elizabethtown | * | ...... |
| O'Neil, Wm. R. | Box 212 | Bedford | * | ...... |
| O'Riley, Martin | 2116 Jefferson | Philadelphia | * | ...... |
| Ormiston, Adam W. | 102 Herman st | G't'n, Phila. | American Vet., 1892 | Philadelphia |
| Orwan, Abraham W. | ...... | New Bl'mfield | * | ...... |
| Osche, Nicholas | ...... | Clearfield | * | ...... |

| | Street. | Town. | College, Graduated. | County, Registered. |
|---|---|---|---|---|
| Osgood, J. R. | ...... | Scranton | * | ...... |
| Osler, W. N. | ...... | Dushore | * | ...... |
| Otstot, Washington | ...... | Shepherdst'n | * | ...... |
| Oyler, J. H. | 14 S. 4th st | Harrisburg | Ontario Vet., 1887 | Dauphin |
| Page, W. H. | ...... | Peckville | * | ...... |
| Paget, H. A. | 415 Spruce st | Scranton | Ontario Vet., 1888 | N'mberland, 1889 |
| Palmer, James B. | ...... | Bernice | * | ...... |
| Palmer, J S. | ...... | Nasby | * | ...... |
| Palmer, Philip C. | ...... | Bryn Mawr | Ontario Vet., 1898 | ...... |
| Park, H W. | ...... | Marietta | * | ...... |
| Patterson, James | ...... | Newportville | * | ...... |
| Patterson, Thomas | ...... | Honesdale | * | ...... |
| Patton, John | ...... | Oakland | * | ...... |
| Pearson, Leonard | 3608 Pine st | Philadelphia | V. D. U. Pa., 1890 | Philadelphia, 1894 |
| Peck, Nicholas L. | ...... | Maytown | * | ...... |
| Peffer, John C. | ...... | Marchand | * | ...... |
| Pegan, Howard F. | Adams st | Cochranton | Ontario Vet., 1897<br>McKillip Vet., 1900 | Crawford, 1901 |
| Pennepacker, Absalom | ...... | Blainsport | * | ...... |
| Penrod, Daniel | ...... | Elderton | * | ...... |
| Permar, G. M. | ...... | Newcastle | * | ...... |
| Peter, Henry W. | Market st | Lewistown | American Vet., 1896 | Mifflin, 1901 |
| Petersheim, S. M. | ...... | Morgantown | * | ...... |
| Pettibon, David | ...... | Burgettstown | * | ...... |
| Phelan, R. M. | ...... | Sharon | * | ...... |
| Phillips, I. B. | 5631 Market st | Philadelphia | * | ...... |
| Phillips, J. R. | 31 Washington | Northeast | Ontario Vet., 1888 | Erie, 1888 |
| Phillips, J. R. | ...... | Library | * | ...... |
| Phillips, Walker S. | 12 N. 3d st | Reading | Boston, 1860 | Berks, 1889 |
| Phillips, Wm. | 95 Union st | Wilkesbarre | * | ...... |
| Phipps, W. P. | 11 N. Canal st | Wilkesbarre | V.D. U. Pa., 1895 | Chester, 1895 |
| Pierce, Jesse W. | 27 W. Main st | Westfield | * | ...... |
| Pierson, R. D. | ...... | Strikersville | * | ...... |
| Pifer, Wm. T. | ..... | Panic | * | ...... |
| Pirman, Henry | 816 Tasker st | Philadelphia | * | ...... |
| Plank, Daniel | ...... | York Springs | * | ...... |
| Plank, John E. | ...... | Idaville | * | ...... |
| Plank, John W. | ...... | Idaville | * | ...... |
| Porter, Edmond C. | 26 S. Mercer st | Newcastle | Ontario Vet., 1891 | Erie, Lawrence, 1891 |
| Potteiger, A. R. | 101 Water st | Selinsgrove | Ontario Vet., 1891 | Lebanon, 1891<br>Snyder, 1893 |
| Powell, Edgar W. | 400 Market st | Oxford | V.D. U. Pa., 1990 | Chester, 1900 |
| Prather, John V. | R.F.D. Route 5 | Titusville | American Vet., 1893 | Crawford, 1893 |
| Prentiss, Samuel M. | ...... | Bloomsburg | * | ...... |
| Price, Jos. F. | Arch st | Newberry | American Vet., 1898 | Lycoming, 1899 |
| Price, Samuel G. | ...... | Doylestown | * | ...... |
| Price, Hubb | ...... | W. Warren | * | ...... |
| Price, S. W. | ...... | Collegeville | * | ...... |
| Prince, Abel | ...... | Cadis | * | ...... |
| Printz, Chas. M. | 2702 W.Fletcher | Philadelphia | * | ...... |
| Pritchard, Gabriel | ...... | Dempseytown | * | ...... |
| Prothers, W. B. | Locust st | Johnstown | N. Y. C. V. S., 1888 | Cambria, 1889 |
| Pryce, David D. | ...... | Ebensburg | * | ...... |
| Pryce, W. D. | ...... | Ebensburg | * | ...... |
| Purcell, P. J. | School st | Bradford | Ontario Vet., 1901 | ...... |
| Purington, B. M | ...... | Corry | * | ...... |
| Purington, M. D. | ...... | Corry | * | ...... |

